# Antimicrobial, Antioxidant, and Antiproliferative Effects of *Coronilla minima*: An Unexplored Botanical Species

**DOI:** 10.3390/antibiotics9090611

**Published:** 2020-09-17

**Authors:** Claudio Ferrante, Paola Angelini, Roberto Venanzoni, Giancarlo Angeles Flores, Bruno Tirillini, Lucia Recinella, Annalisa Chiavaroli, Luigi Brunetti, Sheila Leone, Simonetta Cristina Di Simone, Maria Chiara Ciferri, Gokhan Zengin, Gunes Ak, Luigi Menghini, Giustino Orlando

**Affiliations:** 1 Department of Pharmacy, University “G. d’Annunzio” of Chieti-Pescara, 66100 Chieti, Italy; claudio.ferrante@unich.it (C.F.); lucia.recinella@unich.it (L.R.); annalisa.chiavaroli@unich.it (A.C.); luigi.brunetti@unich.it (L.B.); sheila.leone@unich.it (S.L.); disimonesimonetta@gmail.com (S.C.D.S.); mariachiara.ciferri@outlook.it (M.C.C.); luigi.menghini@unich.it (L.M.); giustino.orlando@unich.it (G.O.); 2 Department of Chemistry, Biology and Biotechnology, University of Perugia, 06100 Perugia, Italy; roberto.venanzoni@unipg.it (R.V.); giancarlo.angelesflores@studenti.unipg.it (G.A.F.); 3 Department of Biomolecular Sciences, University of Urbino, 61029 Urbino, Italy; bruno.tirillini@uniurb.it; 4 Department of Biology, Science Faculty, Selcuk Universtiy, Campus, 42130 Konya, Turkey; akguneselcuk@gmail.com

**Keywords:** *Coronilla minima*, unexplored botanical species, resveratrol, antibacterial effects, antioxidant effects, antiproliferative effects, bioinformatics/docking

## Abstract

*Coronilla* species, belonging to the *Coronilla* genus (Fabaceae), have long been used in traditional medicine for treating cold, diabetes, pain, and as cardiotonics. The goal of the present study was to explore the phytochemical composition and pharmaco-toxicological properties of *C. minima.* In this regard, phenolic content, scavenging/reducing properties and antimicrobial activity toward pathogen bacterial (*Escherichia coli*, *Pseudomonas aeruginosa*, *Bacillus cereus*, *Staphylococcus aureus*) and fungal strains (*Candida albicans*, *C. tropicalis*, *Aspergillus tubigensis* and *A. minutus*) were investigated. Extract effects on human colon cancer HCT116 cell viability were also assayed. Finally, a bioinformatics approach was conducted with the aim to identify putative microbial and human protein targets underlying antibacterial, antimycotic, and antiproliferative effects. Phytochemical investigation suggested that water extract is richer in terms of total flavonoid and phenol content, whereas the hydroalcoholic extract was revealed to be more potent as antioxidant agent. According to bioinformatics analysis, the antibacterial activity of the hydroalcoholic extract could be related to its content in resveratrol. The presence of resveratrol could also explain the hydroalcoholic extract efficacy in reducing HCT116 cell viability. In conclusion, the present study represents the first phytochemical and bio-pharmacological investigation about *C. minima*. Like other plants belonging to the Fabaceae family, *C. minima* revealed a good source of resveratrol, which could explain, albeit partially, the efficacy of the hydroalcoholic extract as antimicrobial, antioxidant, and antiproliferative agent.

## 1. Introduction

The actual urge in searching alternative medications, including nutraceuticals and herbal preparations, that are characterized by a lower grade of side effects compared to the standard of care, has stimulated the botanical and pharmacological research towards plants traditionally used by folk populations to treat inflammatory and infectious disorders [1]. Specifically, an emerging field of research is exploring the rational for using herbal extracts, prepared with traditional and biocompatible solvents (water and hydroalcoholic solution) in the pharmaceutical forms of infusions and decoctions, that would represent the natural link between efficacy and safety, especially in the case of self-medication. Additionally, the present approach could represent a valuable tool to improve and valorize local botanical resources and productive chains [2,3]. *Coronilla* genus (Fabaceae) was originally instituted by Linneus, and subsequently revised more times. Today, the genus includes 11 accepted taxa and is generally distributed in the Mediterranean region, with some species present in Northern Africa, the Middle East, and the Atlantic Islands behind African Coast [4]. Different plant materials of *Coronilla* species (leaves and flowers) have long been used in traditional medicine for treating cold, diabetes and pain, and cardiotonics [5,6]. Polar extracts from *C. varia* have been described as antiproliferative and antibacterial agents, whereas the essential oil was revealed to be superior to extracts as antiradical and enzyme inhibition agents [7,8,9]. Currently, the content of the glycoside hyrcanoside could explain, albeit partially, the cardiotonic and antiproliferative properties of *C. varia* seed extracts [6,10], while terpene compounds such as phytol and γ-terpinene could mediate the antiproliferative effects of the essential oil [9]. *C. minima* is a perennial shrub commonly present in early successional stages with a Mediterranean area distribution. It produces a dry-fruit legume containing up to five seeds that are dispersed by gravity or by ants [11]. The primary metabolite profile of *C. minima* aerial parts was investigated for determining protein content and amino acid profile as well as lipid and fatty acid patterns within a comparative study including ten legume producing species. The study also focused on the potential nutritional role in feeding supplementation and highlighted the presence of amino acids and unsaturated fatty acids, mainly represented by palmitic acid [12]. To the best of our knowledge, *C. minima* was never investigated for secondary metabolite composition or pharmaco-toxicological profile. In this regard, the goal of the present multidirectional investigation was to explore the phytochemical composition, antioxidant and bio-pharmacological properties of water and hydroalcoholic extracts from aerial parts. Specifically, phytochemical analysis was conducted through colorimetry, mass spectrometry and high performance liquid chromatography tecniques. Antimicrobial activity was studied on multiple pathogen bacterial and fungal strains, namely *Escherichia coli*, *Pseudomonas aeruginosa*, *Bacillus cereus*, *Staphylococcus aureus*, *Candida albicans*, *C. tropicalis*, *Aspergillus tubigensis*, and *A. minutus*. Extract effects on human colon cancer HCT116 cell viability were also investigated. Finally, a bioinformatics approach was conducted with the aim to identify putative microbial and human protein targets underlying the observed bio-pharmacological effects. Emphasis was given to the predicted interactions between extract compounds and bacterial and human proteins involved in oxidative metabolism, namely superoxide dismutase, nitric oxide, cytochrome P450, cyclooxygenase, and carbonic anhydrase.

## 2. Materials and Methods

### 2.1. Plant Material

Aerial parts of *Coronilla minima* L. were collected in June 2018 in Abruzzo (GPS coordinates 42.279252, 13.462557) from wild plants in full bloom. Botanical species was identified by Luigi Menghini (Professor of Botany at the Department of Pharmacy “G. d’Annunzio” University). Fresh plant material was dried in a ventilated oven (40 °C) until it reached a constant weight. Dry plant material was grinded to obtain uniform 20 mesh granulometry and finally stored in sealed plastic bags in the dark until extraction.

### 2.2. Extraction

Two extraction methods (sonicator-assisted and Soxtec extraction) in the presence of heating were used to prepare water and hydroalcoholic *C. minima* extracts. Details about optimized extraction conditions are detailed in Table 1 and the efficiency was calculated as dry extract yield, total phenol, and flavonoid recovery. Extracts were taken to dryness under reduced pressure (Speedvac) and resuspended just before bio-pharmacological tests. Dry extracts were stored in the dark at 4 °C, avoiding light exposure.

### 2.3. Estimation of Total Phenolic/Flavonoid Compounds and Antioxidant Activity

Colorimetric Folin–Ciocalteu and AlCl_3_ methods were selected to quantify total phenols and flavonoids, respectively [13,14]. Total phenols and flavonoids were expressed as µg gallic acid/g dry extract and µg rutin/g of dry extract, respectively. Antioxidant activity was evaluated through colorimetric α, α-diphenyl-β-picrylhydrazyl (DPPH), 2,2-azinobis-(3-ethylbenzthiazoline-6-sulfonic acid) (ABTS), cupric reducing antioxidant capacity (CUPRAC), ferric reducing antioxidant power (FRAP), phosphomolybdenum and metal chelating assays [15,16].

### 2.4. Mass Spectrometry and High Performance Liquid Chromatography (HPLC) Analysis

*C. minima* extracts were qualitatively analyzed with mass spectrometry (MS) (Advion expression compact mass spectrometer (CMS), Advion, Ithaca, NY, USA) in negative ion mode [mass-to-charge ratio (*m*/*z*) scan mode: 119–556)]. Signal identification was realized through comparison with MS spectra present in the recognized database MassBank Europe (https://massbank.eu/MassBank/). Quantitative determination of phenolic compounds identified by MS analysis was performed through reversed-phase HPLC-fluorimeter (MOD. 1525/2475, Waters Corporation, Milford, MA, USA). The analytical conditions were selected according to literature [16,17]. The HPLC stationary phase was a C18 reversed-phase column (AcclaimTM 120, 3 μm, 2.1 × 100 mm, Dionex Corporation, Sunnyvale, CA, USA). The separation was carried out in gradient elution mode: methanol/acetic acid/water (10:2:88, *v*/*v*) as solvent A, and methanol/acetic acid/water (10:2:88, *v*/*v*) as solvent B. The fluorimetric detection conditions were λex = 278 nm and λem = 360 nm wavelengths.

### 2.5. Antimicrobial Susceptibility Testing

*C. minima* water and hydroalcoholic extracts were tested for antimicrobial activity against *Pseudomonas aeruginosa* (ATCC 15442), *Escherichia coli* (ATCC 10536), *Staphylococcus aureus* (ATCC 6538), *Bacillus cereus* (ATCC 12826), *Candida albicans* (YEPGA 6183), *C. tropicalis* (YEPGA 6184), *Aspergillus tubingensis* (PeruMicA 21), and *A. minutus* (PeruMicA 22). The antimicrobial activity of the aforementioned extracts were compared to ciprofloxacin (Sigma-Aldrich, Milan, Italy) and fluconazole (Sigma-Aldrich, Milan, Italy), which are used as antibacterial and antimycotic reference drug, respectively [18,19].

### 2.6. Antibacterial Activity Assay

Minimum inhibitory concentration (MIC) was calculated according to the broth dilution method M07-A9 [20]. To further assess the viability of bacterial cells at MIC end-points, the tetrazolium salt assay optimized by Sabaeifard and collaborators (2014) was used to determine the viability of bacterial cells at MIC end-points [21].

### 2.7. Antifungal Activity Assay

The susceptibility test against fungal strains was conducted as previously reported [19,20,22,23]. MIC end-points (µg mL^−1^) were calculated after 24 h (for *Candida albicans* and *C. tropicalis*) and 48 h (for *A. tubingensis* and *A. minutus*) [19,20]. For *C. minima* extracts, the MIC end-points were the lowest concentrations showing total growth inhibition [24].

### 2.8. Eco-Toxicological Assays

The biocompatibility of *C. minima* extracts (0.1–40 mg/mL) was evaluated through independent eco-toxicological tests, namely allelopathy and brine shrimp lethality test. The first evaluated the capability of *C. minima* extracts to modify the seedling germination of lettuce varieties Lollo bionda, Iceberg and Trocadero. The second assay measures the survival of brine shrimp (*Artemia salina*) cysts after challenging with extracts. The complete description of each test is reported in previous papers [25,26].

### 2.9. Cell Culture

HCT116 cell line (ATCC^®^ CCL-247™) was cultured as previously reported [27]. *C. minima* extract cytotoxicity was determined via the 3-(4,5-dimethylthiazol-2-yl)-2,5-diphenyltetrazolium bromide (MTT) test in basal condition and after challenging with serotonin (5-HT) at 1 ng/mL concentration.

### 2.10. Bioinformatics

Microbial and human proteins targeted by *C. minima* extracts were predicted using bioinformatics platforms STITCH (http://stitch.embl.de/cgi/network.pl) and SwissTargetPrediction (http://www.swisstargetprediction.ch/). Docking calculations were conducted through the Autodock Vina of PyRx 0.8 software. Crystal structures of target proteins were derived from the Protein Data Bank (PDB) with PDB IDs as follows: 1XRE (superoxide dismutase-A (SOD-A) from *B. cereus*), 3TQJ (superoxide dismutase-B (SOD-B) from *B. cereus*), 5JQV (Cytochrome P450 (CypD) from *B. cereus*), 2FLQ (nitric oxide synthase (NOS) from *B. cereus*), 1EQG [cyclooxygenase-1 (COX-1)], and 5FL6 [carbonic anhydrase IX (CA-IX)]. Discovery studio 2020 visualizer was employed to investigate the protein-ligand non-bonding interactions.

### 2.11. Statistical Analysis

Statistical analysis was conducted through GraphPad Prism version 5.01 for Windows (GraphPad Software, San Diego, CA, USA). Experimental data were derived from three experiments conducted in triplicate and were analyzed through analysis of variance (ANOVA) followed by Tukey’s post-hoc test. Results were considered significant for values of *p* < 0.05.

## 3. Results and Discussion

**Figure 1:**
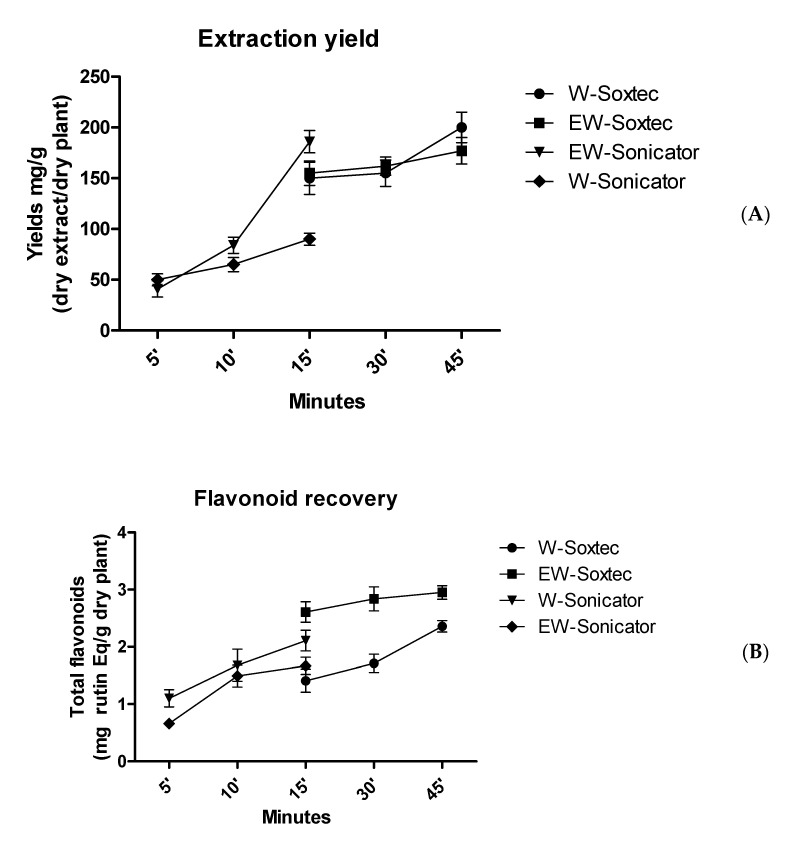

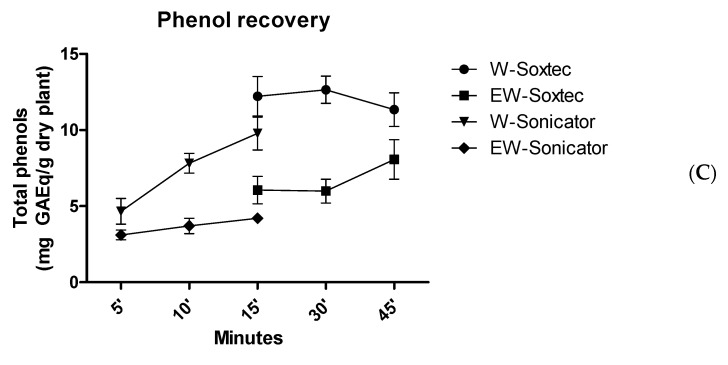
Optimization of *C. minima* extraction with water and hydroalcoholic solution following sonication- and Soxtec-assisted extractive methods. (**A**) Dry extract yield; (**B**) total phenol yield; (**C**) total flavonoid yield.

**Figure 2:**
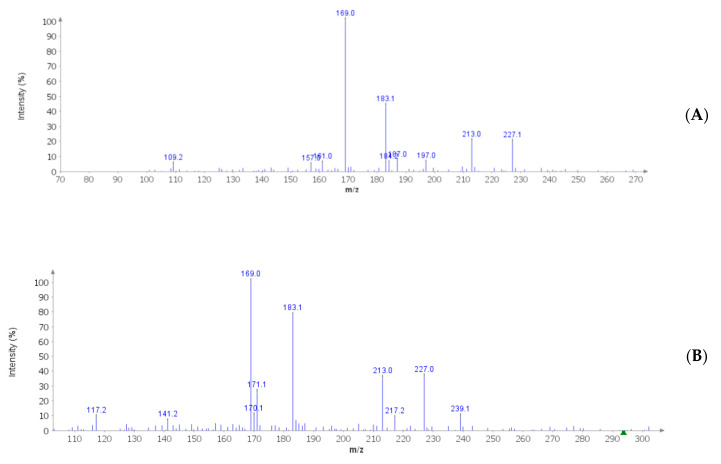
Qualitative mass spectrometry (MS) analysis of *C. minima* extracts yielding gallic acid and resveratrol signals at mass-to-charge ratio (*m*/*z*) 169 and 227, respectively. Data were confirmed by comparison with gallic acid and resveratrol MS spectra collected by MassBank Europe (https://massbank.eu/MassBank/). (**A**) MS spectrum of *C. minima* water extract. (**B**) MS spectrum of the *C. minima* hydroalcoholic extract.

The pharmacognostic characterization of *C. minima* started from the optimization of the extraction procedures. In this regard, laboratory Soxtec- and sonicator-assisted methods were chosen in order to mimic the home-made preparations through the use of biocompatible solvents, namely water and hydroalcoholic solution [ethanol/water 50:50 (*v*:*v*)]. Details about extraction conditions are depicted in Table 1. The extraction efficiency was evaluated in terms of dry extract yield, total phenol, and flavonoid recovery (Figure 1A–C). Regarding the Soxtec-assisted method, there were no significant differences between water and hydroalcoholic solvent with regard to the extraction yield, which was also similar to that of the hydroalcoholic extract prepared via sonication. The comparative study also permitted the optimization of the time of extraction, that was concluded after 15 min with both methods. Conversely, the water extract prepared through sonication showed the lowest extraction yield. In parallel, phenol and flavonoid recovery was evaluated as gallic acid equivalents (GAE)/dry plant and rutin equivalents (Eq)/mg extract, respectively. As depicted in Figure 1B,C, the water extract displayed the highest yield of these secondary metabolites. On the other hand, the highest intrinsic antiradical activity was shown by hydroalcoholic extract in DPPH and linoleic acid assays (Table 2), thus suggesting that other compounds could influence the extract scavenger properties. Considering the results of extraction experiments, sonication was chosen as the elective method for mimicking home-made preparations. In this regard, the following phytochemical and bio-pharmacological assays were conducted on water and hydroalcoholic extracts prepared with this procedure. Specifically, extracts were qualitatively analyzed by negative ion mode MS analysis (Figure 2A,B), which confirmed the presence of gallic acid (*m*/*z*: 169) and resveratrol (*m*/*z*: 227). As a further analytical investigation, water and hydroalcoholic extracts were also assayed through HPLC coupled with fluorimetric detection for gallic acid and resveratrol quantification (Table 3). In both extracts, resveratrol content was consistent with its presence in other plants belonging to the Fabaceae family [28]. Additionally, its identification was also demonstrated in multiple plant families and species, namely *Arachis hypogea* L., *Vitis vinifera* L., *Pinus sylvestris* L., *Rheum palmatum* L., *Gardenia jasmonoides Ellis*, *Phlomis umbrosa* Turgi, *Polygala tenuifolia* Willd., and *Angelica dahurica* Benth. et Hook [29], thus increasing the interest in investigating the presence of resveratrol in plant extracts. This is obviously due to resveratrol recognized intrinsic antioxidant effects and ability to modulate cell pathways controlling oxidative response [30,31]. Consistent with colorimetric assays, the water extract showed the highest amount of gallic acid, which is a marker of phenolic composition. On the other hand, in the hydroalcoholic extract, the resveratrol level was about 20-fold higher compared to that of the water extract. This could be partially related to the higher solubility of resveratrol in hydroalcoholic solution, thus further substantiating the better scavenger properties of the hydroalcoholic extract compared to the water extract.

**Figure 3:**
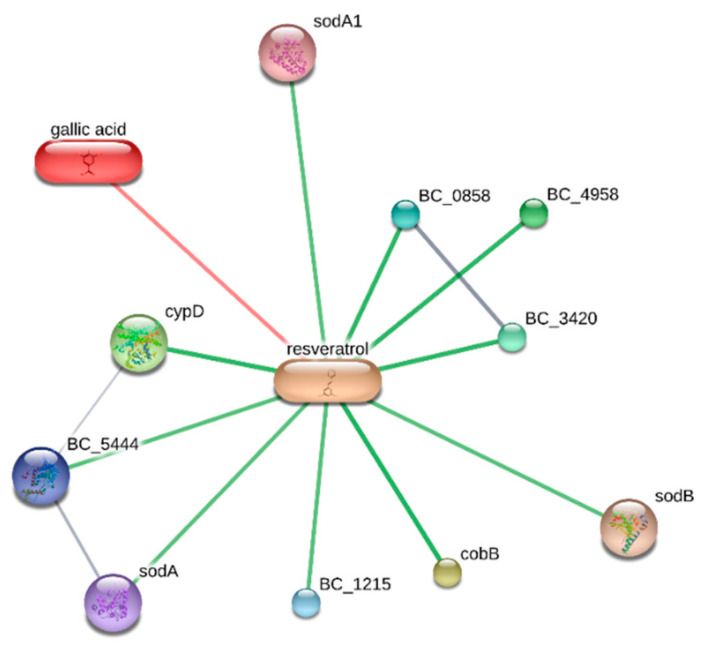
Targets–components analysis related to the putative bacterial (*B. cereus*) proteins that are principally targeted by resveratrol.

**Figure 4:**
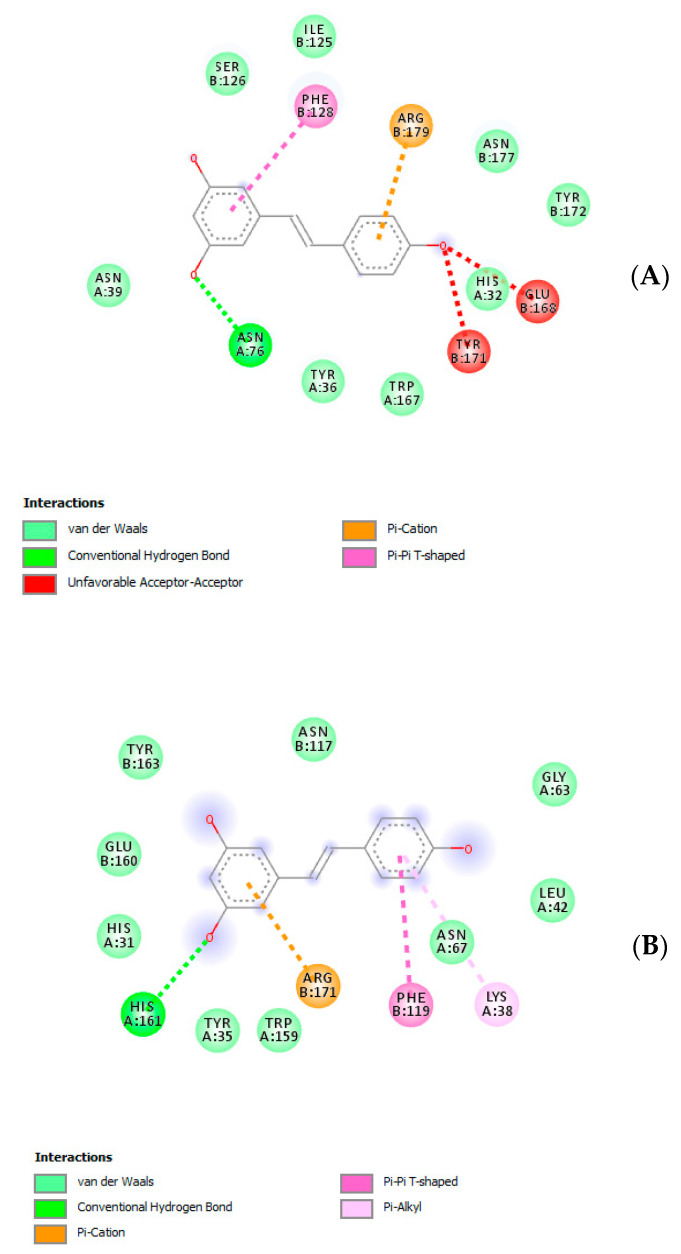

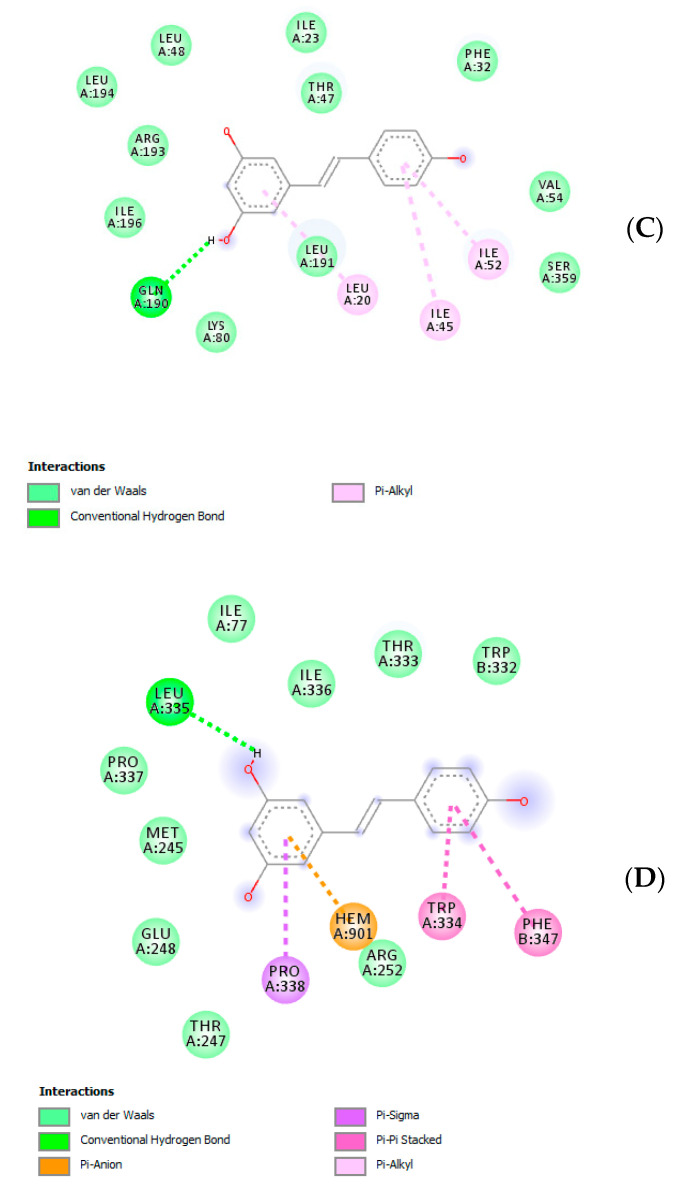
(**A**) Putative interactions between resveratrol and *B. cereus* superoxide dismutase A (SOD-A; PDB: 1XRE). Free energy of binding (ΔG) and affinity (Ki) are −7.0 kcal/mol and 7.5 µM, respectively; (**B**) Putative interactions between resveratrol and *B. cereus* superoxide dismutase B (SOD-B; PDB: 3TQJ). Free energy of binding (ΔG) and affinity (Ki) are −7.2 kcal/mol and 5.4 µM, respectively; (**C**) Putative interactions between resveratrol and *B. cereus* cytochrome P450 (CypD; PDB: 5JQV). Free energy of binding (ΔG) and affinity (Ki) are −7.4 kcal/mol and 3.8 µM, respectively; (**D**) Putative interactions between resveratrol and *B. cereus* nitric oxide synthase (BC_5444; PDB: 2FLQ). Free energy of binding (ΔG) and affinity (Ki) are −7.7 kcal/mol and 2.3 µM, respectively.

The observed scavenging/reducing properties and phenolic content of extracts support bio-pharmacological investigations in order to unravel phytotherapy applications. Initially, the extracts were tested in different ecotoxicological assays, namely allelopathy and brine shrimp lethality test, in order to define biocompatibility limits. Specifically, in both assays, water and hydroalcoholic extracts were studied in a wide concentration range (0.1–40 mg/mL). Regarding the allelopathy assay, extracts did not significantly affect the growth of lettuce varieties *Lollo bionda,* Iceberg and Trocadero, yielding seedling germinations ≥90% compared to untreated control group, at any given extract concentration. On the other hand, the brine shrimp lethality test showed extracts’ biocompatibility at lower concentrations, giving LC_50_ values < 40 mg/mL. According to the present findings, biocompatibility extract concentrations should be at least 10-fold lower compared to LC_50_ values observed in the brine shrimp test [26]. Considering these results, antimicrobial effects of water and hydroalcoholic extracts were studied against selected pathogen bacterial and fungi strains that are involved in numerous disorders, including colon inflammation [26]. As depicted in Table 4, both extracts were equally effective against *C. albicans* and *A. minutus,* although the hydroalcoholic extract showed lower MIC values. This result could be related only partially to the phenol and flavonoid content displayed by both extracts [26]. In fact, regarding the antibacterial activity (Table 5), the water extract, despite showing the highest total phenol and flavonoid content, was completely ineffective against *B. cereus*, *E. coli*, *S. aureus* and *P. aeruginosa.* Conversely, the hydroalcoholic extract was effective against all four selected bacterial strains with MIC values in the range of 3.57–7.14 µg/mL. In this regard, *B. cereus* was the most sensitive strain to the cytostatic properties of the extract. The extract MIC values were also in the range of biocompatibility. Therefore, a bioinformatics approach was conducted through the stitch platform (http://stitch.embl.de/) with the aim to predict putative *B. cereus* targets underlying the observed effects. Considering the composition of the hydroalcoholic extract, a components–targets analysis was conducted on gallic acid and resveratrol and the results are depicted in Figure 3. It is of noteworthy interest that resveratrol was predicted to be the phytocompound in the prominent position in the targets–components analysis. Resveratrol could interact with multiple proteins involved in bacterial oxidative metabolism including superoxide dismutases (SOD-A and SOD-B), nitric oxide synthase (BC_5444), and NADPH-cytochrome P450 reductase (CypD). In this regard, docking analysis showed a good affinity of resveratrol toward all docked proteins (Figure 4A–D), with Ki values in the micromolar range. This further corroborates the antimicrobial effects exerted by hydroalcoholic extract. This is also consistent with antibacterial effects exerted by polar extracts prepared from *C. varia* [7].

**Figure 5:**
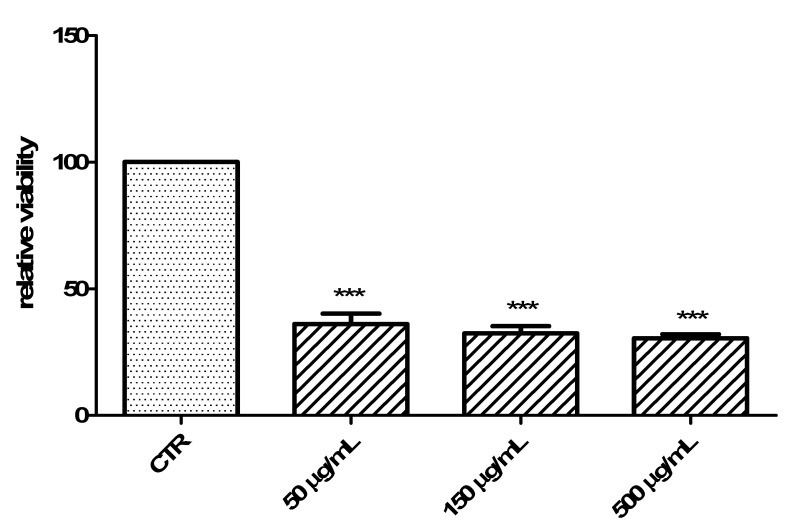
Antiproliferative effect of *C. minima* hydroalcoholic extract 50–500 µg/mL on human colon cancer HCT116 cell viability [3-(4,5-dimethylthiazol-2-yl)-2,5-diphenyltetrazolium bromide (MTT) test] in basal conditions. ANOVA, *p* < 0.0001; *** *p* < 0.001 vs. CTR group.

**Figure 6:**
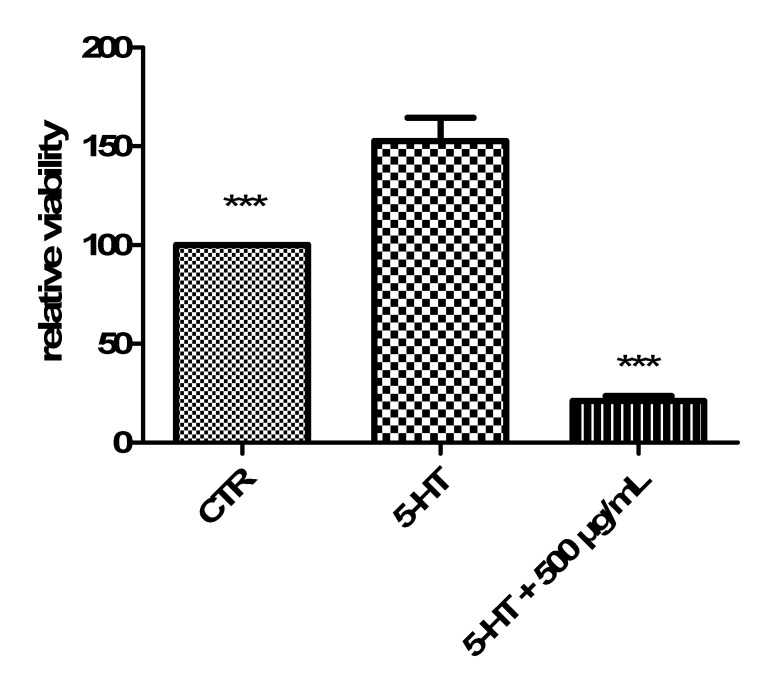
Antiproliferative effect of *C. minima* hydroalcoholic extract 500 µg/mL on human colon cancer HCT116 cell viability [3-(4,5-dimethylthiazol-2-yl)-2,5-diphenyltetrazolium bromide (MTT) test] induced by the mitogen stimulus 5-HT (1 ng/mL). ANOVA, *p* < 0.0001; *** *p* < 0.001 vs. 5-HT group.

Usta and colleagues (2014) also studied antitumor activities of the same polar extracts [7]. On the other hand, those effects were demonstrated using the potato disc method, which has a limit of being a preliminary antitumoral screening, with partial translational significance toward the human model [32]. Therefore, in the present study, we investigated the antiproliferative effects of the hydroalcoholic extract toward the human colon cancer HCT116 cell line. In this context, we explored the influence of cell exposure to the extract on basal and serotonin (5-HT)-induced viability [27]. 5-HT, besides being a well-known and multifunctional neurotransmitter in the brain, is also a crucial pro-inflammatory and mitogen factor in the gut, with the capacity to increase the viability of several tumoral cell lines including HCT116 [33]. The hydroalcoholic extract (50–500 µg/mL) displayed a significant and concentration-independent inhibition of HCT116 cell viability in basal conditions (Figure 5). The extract was also able to blunt the 5-HT-induced HCT116 cell viability (Figure 6) with an efficacy comparable to that of the anticancer drug daunorubicin [25].

**Figure 7:**
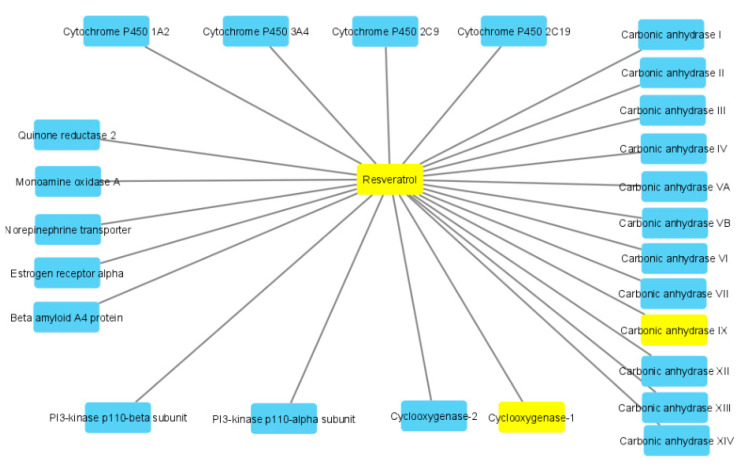
Targets–components analysis related to high probable putative interactions of resveratrol with multiple human proteins including cyclooxygenases and carbonic anhydrases.

**Figure 8:**
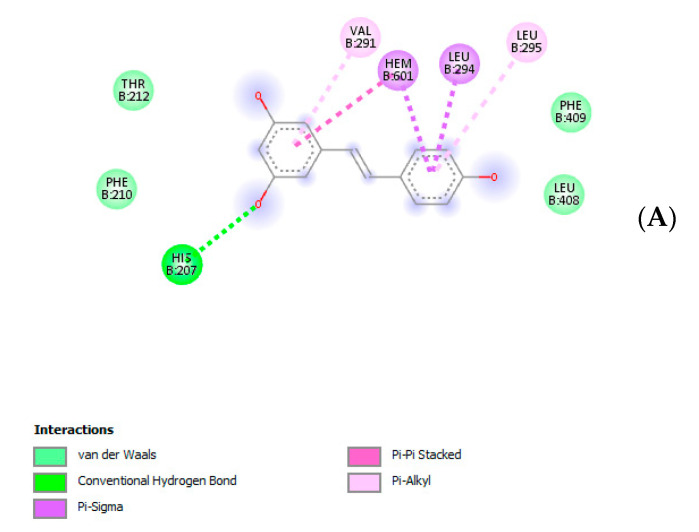

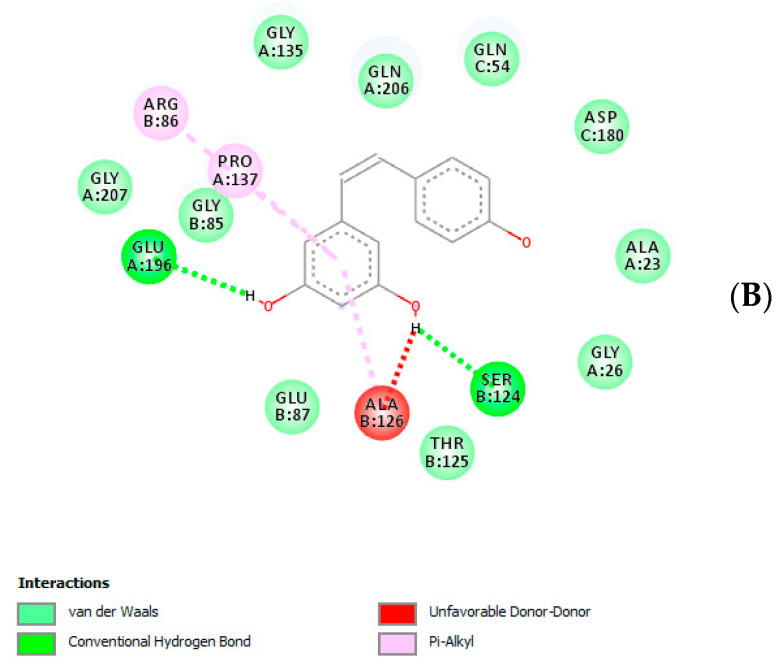
(**A**) Putative interactions between resveratrol and cyclooxygenase-1 (COX-1; PDB: 1EQG). Free energy of binding (ΔG) and affinity (Ki) are −7.8 kcal/mol and 1.9 µM, respectively. (**B**) Putative interactions between resveratrol and carbonic anhydrase-IX (CA-IX; PDB: 5FL6). Free energy of binding (ΔG) and affinity (Ki) are −7.3 kcal/mol and 4.5 µM, respectively.

Given the observed reduction of HCT116 cell line viability, a further bioinformatics approach was conducted in order to predict human protein targets putatively able to interact with the extract resveratrol pool. The phytocompound structure was run on the bioinformatics platform SwissTargetPrediction (http://www.swisstargetprediction.ch/), which predicted a high probability of interaction between resveratrol and multiple enzyme families, including carbonic anhydrases (CAs), cyclooxygenases (COXs) and cytochromes (Figure 7). Considering these results, docking calculations were carried out to predict the affinity of resveratrol toward CA-IX and COX-1 (Figure 8A,B), whose levels were found to be upregulated in colon cancer [34,35]. The results of in silico experiments suggest the capability of resveratrol to interact with these enzymes in the micromolar range. The docking calculations were also consistent with multiple in vitro studies highlighting inhibitory effects of resveratrol and its analogs on colon cancer cell viability in both basal and pro-inflammatory conditions [36,37,38].

In order to improve our knowledge about the bio-pharmacological properties of the hydroalcoholic extract, colorimetric assays were also performed with the aim to evaluate scavenging/reducing and metal chelating properties (Table 6). This approach is believed to provide more accurate and comprehensive informations regarding the antioxidant potential of herbal extracts [39,40,41]. Additionally, concomitant antiradical and antiproliferative effects were reported for different natural products and herbal extracts [42,43]. The observed scavenging/reducing effects of *C. minima* hydroalcoholic extracts were consistent with the inhibition of colon cancer HCT116 cells, especially when cells were exposed to 5-HT challenge, which is able to increase oxidative stress and induce HCT116 cell viability [27,33]. Finally, *Coronilla* species were found to be sources of different classes of secondary metabolites with putative antiproliferative effects, including cardenolides [7,9,10]. In this regard, further studies need to better characterize the qualitative composition of the phytocomplex.

## 4. Conclusions

In conclusion, the present study represents the first phytochemical and bio-pharmacological investigation about *C. minima*. Specifically, all tested extracts displayed an antimycotic effect against *C. albicans* and *A. minutes.* On the other hand, the sole hydroalcoholic extract was effective in inhibiting *B. cereus* growth. Like other plants belonging to the Fabaceae family, *C. minima* revealed a good source of phenolic compounds, including resveratrol. This could explain, albeit partially, the efficacy of the hydroalcoholic extract as antimicrobial, antioxidant and antiproliferative agent. Overall, the present results support further investigation for improving the phytochemical and pharmacological characterization of *C. minima*, also taking into consideration alternative experimental and therapeutic targets. In this context, the next step will be a deeper phytochemical investigation to complete the characterization of the plant phytocomplex. In parallel, pharmacological studies will be conducted in order to unravel the mechanism of action underlying the observed antimicrobial and antiproliferative effects.

## Figures and Tables

**Table 1:** Extraction parameters.

Soxtec-Assisted Extraction: Extraction Time (Immersion-Rinse)	45 min–15 min	30 min–15 min	15 min–15 min
H_2_O (200 °C) *			
Et-OH/H_2_O 50:50 (190 °C) *			
Sonication-assisted extraction: extraction time	15 min	10 min	5 min
H_2_O (60 °C) *			
Et-OH/H_2_O 50:50 (25 °C) *			

* Temperature values indicate instrumental setting.

**Table 2:** Phytochemical analysis.

Extracts	Total Phenolic Content (mg GAE/mL ± SD)	Total Flavonoid Content (mg RE/mL ± SD)	DPPH Test IC_50_ (µg/mL ± SD)	Linoleic Assay IC_50_ (µg/mL ± SD)
Water	128.23 ± 7.56	100.42 ± 6.09	26.43 ± 4.20	5.44 ± 0.54
EtOH–Water	61.04 ± 9.18	8.95 ± 2.05	100.49 ± 7.41	54.93 ± 3.99

Values expressed are means ± S.D. of three parallel measurements. GAE: Gallic acid equivalent; RE: Rutin equivalent.

**Table 3:** Phenolic compound levels (mg/g extract) in water and hydroalcoholic extracts from *C. minima*.

Solvents	Gallic Acid	Resveratrol
Water	722.89 ± 65.06	3.18 ± 0.32
Hydroalcoholic solution	227.25 ± 9.11	61.82 ± 6.99

**Table 4:** Minimum inhibitory concentration (MIC) of plant extract toward selected yeasts and filamentous fungal strains.

Minimum Inhibitory Concentration (MIC) [µg (Dry Weight) Extract mL^−1^] *						
Plant Species	Reference Antimycotic Drug	Extract Typology	*C. albicans* (YEPGA 6183)	*C. tropicalis* (YEPGA 6184)	*A. tubingensis* (PeruMycA 21)	*A. minutus* (PeruMycA 22)
*Coronilla minima*		H_2_O	11.34 (9–18)	>18	>18	14.28 (9–18)
		H_2_O:EtOH (1:1)	7.14 (4.5–9)	>18	>18	11.34 (9–18)
	Fluconazole	-	2	4	>16	>16

* MIC values (µg/mL) are reported as geometric means of three independent replicates (*n* = 3); MIC range concentrations are reported within brackets.

**Table 5:** Minimum inhibitory concentration (MIC) of plant extracts toward selected bacterial strains.

Minimum Inhibitory Concentration (MIC) [µg (Dry Weight) extract ml^−1^] *						
Plant Species	Reference Antibacterial Drug	Extract Typology	*B. cereus* (ATCC 12826)	*S. aureus* (ATCC 6538)	*E. coli* (ATCC 10536)	*P. aeruginosa* (ATCC 15442)
*Coronilla minima*		H_2_O	>18	>18	>18	>18
		H_2_O:EtOH (1:1)	3.57 (2.25–4.50)	7.14 (4.5–9)	7.14 (4.5–9)	7.14 (4.5–9)
	Ciprofloxacin	-	<0.12	0.62 (0.98–0.49)	<0.12	1.23 (1.95–0.98)

* MIC values (µg/mL) are reported as geometric means of three independent replicates (*n* = 3); MIC range concentrations are reported within brackets.

**Table 6:** Scavenging/reducing and metal chelating properties of the *C. minima* hydroalcoholic extract.

Assays	Results
CUPRAC (mg TE/g)	62.28 ± 0.22
FRAP (mg TE/g)	38.88 ± 1.32
DPPH (mg TE/g)	19.98 ± 0.76
ABTS (mg TE/g)	61.39 ± 0.30
Phosphomolybdenum (mmol TE/g)	0.72 ± 0.07
Metal chelating activity (mg EDTAE/g)	35.83 ± 0.08

Values are reported as mean ± SD of three parallel experiments conducted in triplicate.
